# Metformin treatment in heart failure with preserved ejection fraction: a systematic review and meta-regression analysis

**DOI:** 10.1186/s12933-020-01100-w

**Published:** 2020-08-05

**Authors:** Amera Halabi, Jonathan Sen, Quan Huynh, Thomas H. Marwick

**Affiliations:** 1 (Dept) Imaging Research, Baker Heart and Diabetes Institute, PO Box 6492, 75 Commercial Road, Melbourne, VIC 3004 Australia; 2 School of Public Health and Preventive Medicine, Monash University, 553 St Kilda Road, Melbourne, VIC 3004 Australia; 3 (Dept) Imaging Research, Menzies Institute for Medical Research, 17 Liverpool Street, Hobart, TAS 7000 Australia; 4 Faculty of Medicine, Dentistry and Health Sciences, The University of Melbourne, 207 Bouverie Street, Parkville, VIC 3010 Australia

**Keywords:** Diabetes mellitus, Metformin, Heart failure, Ejection fraction

## Abstract

**Background:**

Observational series suggest a mortality benefit from metformin in the heart failure (HF) population. However, the benefit of metformin in HF with preserved ejection fraction (HFpEF) has yet to be explored. We performed a systematic review and meta-analysis to identify whether variation in EF impacts mortality outcomes in HF patients treated with metformin.

**Methods:**

MEDLINE and EMBASE were searched up to October 2019. Observational studies and randomised trials reporting mortality in HF patients and the proportion of patients with an EF > 50% at baseline were included. Other baseline variables were used to assess for heterogeneity in treatment outcomes between groups. Regression models were used to determine the interaction between metformin and subgroups on mortality.

**Results:**

Four studies reported the proportion of patients with a preserved EF and were analysed. Metformin reduced mortality in both preserved or reduced EF after adjustment with HF therapies such as angiotensin converting enzyme inhibitors (ACEi) and beta-blockers (β = − 0.2 [95% CI − 0.3 to − 0.1], *p *= 0.02). Significantly greater protective effects were seen with EF > 50% (*p *= 0.003). Metformin treatment with insulin, ACEi and beta-blocker therapy were also shown to have a reduction in mortality (insulin *p *= 0.002; ACEi *p *< 0.001; beta-blocker *p *= 0.017), whereas female gender was associated with worse outcomes (*p* < 0.001).

**Conclusions:**

Metformin treatment is associated with a reduction in mortality in patients with HFpEF.

## Background

Heart failure (HF) with preserved ejection fraction (HFpEF) is a distinct phenotype hallmarked by clinical signs and symptoms of HF coupled with a normal ejection faction (EF ≥ 50%) and evidence of increased left ventricular (LV) pressures and impaired LV filling on echocardiography [1–3]. HFpEF accounts for almost half of the cases of HF and carries an equally poor prognosis to those with HF with reduced ejection fraction (HFrEF), with an estimated 4-year mortality rate of 32% [4]. In contrast to HFrEF, where several therapies have shown good long-term morbidity and mortality outcomes, despite multiple aetiologies leading to the same pathophysiological end-point [1–3, 5], effective therapy options for HFpEF have yet to be established. In part, this is because HFpEF is a heterogenous condition, with phenotypic clusters based on age, gender and comorbid illnesses such as obesity, type 2 diabetes mellitus (T2DM) and hypertension [6, 7]. This ultimately leads to dysfunctional metabolic pathways and mechanics within the myocardium resulting in the condition [6, 8]. Therefore, establishing therapy that targets these phenotypes may be the means by which HFpEF therapy evolves [6].

Metformin is a common anti-diabetic drug with both systemic and cardioprotective benefits in addition to its hypoglycaemic effect [9, 10]. At the cellular level metformin activates adenosine monophosphate-activated protein kinase (AMPK) an important regulator of several metabolic pathways resulting in enhanced glucose utilisation, reduction of protein synthesis and improvement of mitochondrial function [11–13]. Furthermore, metformin has been shown to reduce collagen accumulation and potentially reduce LV hypertrophy and improve diastolic function in the diabetic myocardium [14]. Several observational series have shown a reduction in mortality in the HF population [15, 16]. Its mortality benefit in the HFpEF population however has yet to be explored. We performed a systematic review and meta-regression analysis to identify whether variations in ejection fraction (EF) impact mortality outcomes in HF patients treated with metformin.

## Methods

This systematic review was undertaken in accordance with the Preferred Reporting Items for Systematic Reviews and Meta-Analysis (PRISMA) statement [17]. The review was registered with the PROSPERO International prospective register of systematic reviews (ID CRD42019133780) in September 2019.

### Literature search

MEDLINE (1946 to October 2019) and EMBASE (1947 to October 2019) electronic databases using Ovid^®^ were searched for randomised controlled trials and observational studies that assessed the impact of metformin therapy on mortality outcomes in adult HF patients (aged over 18 years). Common search terms included (‘heart failure’ or ‘cardiomyopathies’), (‘diastolic heart failure’ or ‘preserved ejection fraction’), (‘metformin’ or ‘biguanide’) and (‘mortality’ or ‘death’). The full MEDLINE and EMBASE search strategies are detailed in the Additional file 1: Appendix S1. Reference mining of articles in the full-text review was undertaken as well as grey literature searching. Searches were restricted to human studies and those reported in the English language.

### Study selection

Two reviewers (A.H. and J.S.) independently undertook abstract screening and included studies reporting mortality outcomes in HF patients treated with metformin. Studies were divided as preserved or reduced EF; with the preserved group further subdivided into those that reported proportion of patients with EF ≥ 50% or EF ≥ 40%. and reported the proportion of patients with an EF ≥ 50%. Studies were excluded if [1] diagnosis of HF was based purely on hospital discharge codes and no information specifically pertaining to EF and mortality outcomes were recorded, and [2] quality of methodology was not able to be critically appraised, for example in conference abstracts and unpublished studies. After exclusions based on title and abstract review, two investigators (A.H. and J.S.) independently undertook full text reviews for eligibility. Reference searching of review articles was undertaken to search for additional studies, however review articles were not formally included in the systematic review. Covidence^®^ (Melbourne, Australia) software was used to track articles in the systematic review process. Conflicts were resolved by a third reviewer (T.H.M.).

### Data extraction

Data extraction from eligible studies was undertaken independently by two researchers (A.H. and J.S.). Data was extracted on study design and characteristics, including year of publication, number of subjects, gender, duration of follow-up, medical history (including history of coronary artery disease (CAD), hypertension and peripheral vascular disease (PVD)), baseline treatment with cardio-protective (angiotensin converting enzyme inhibitor (ACEi)/angiotensin receptor blocker (ARB), beta-blocker), and anti-diabetic medications (insulin and sulfonylurea therapy) and EF ≥ 50% on echocardiography. Hazard ratios (HR) with associated 95% confidence intervals (95% CI) on mortality outcomes stratified by the presence or absence of metformin treatment were extracted.

### Quality and risk of bias assessment

Quality and risk of bias was assessed using the Newcastle–Ottawa quality assessment scale for cohort studies. This scale assesses the quality of a study based on patient selection, comparability and outcome. Included studies were ranked as good, fair or low quality as outlined in Additional file 1: Appendix S2.

### Statistical analysis

Proportion of individuals within each subgroup are expressed as a percentage (%). The meta-analysis was performed using maximally adjusted HR and 95% CI to obtain an overall effect size using a random-effects model. Heterogeneity between studies was tested for mortality outcomes using the Chi square test with a *p* value < 0.05 being statistically significant. An I^2^ statistic was generated with > 20% heterogeneity considered significant. A sampling weight was assigned to adjust for differences in study size contributions between each study by calculating the proportion of patients within each sub-group based on the total number of individuals included in the final meta-analysis. Meta-regression models were then performed to determine the interaction between the presence **or** absence of metformin therapy on mortality outcomes in each sub-group. All statistical analyses were performed using STATA software (StataCorp LLC 2019, v.16.0, College Station, TX, USA).

## Results

### Study selection

**Fig. 1:**
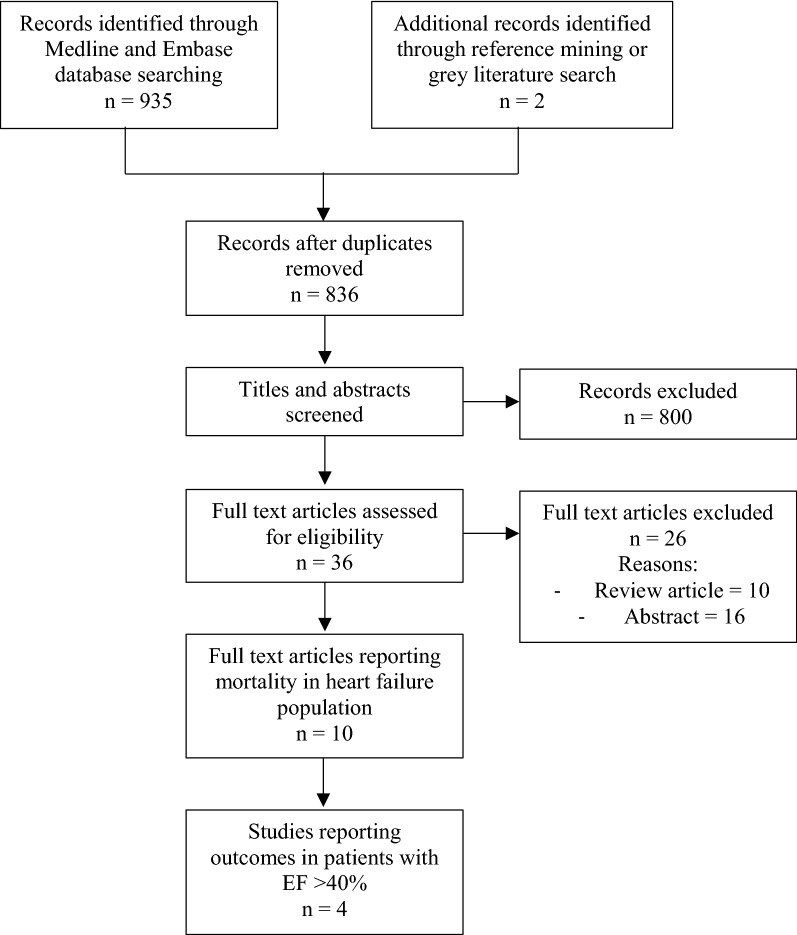
Flow diagram showing literature search outcome and study selection

There were 836 studies identified in the search strategy on mortality outcomes in HF patients treated with metformin, with 10 undergoing full-text review (Fig. 1). Of these, 4 reported the total proportion of patients at baseline with an EF ≥ 50% (Table 1) and were included in the final analysis [18–21]. Of the 6 studies excluded from the final analysis, 1 study excluded HF patients with an LVEF ≥ 40% [22] and 5 studies diagnosed HF at baseline based on hospital discharge diagnosis or International Classification of Diseases (ICD) or Medicare codes and did not report EF data [23–27] as outlined in the Additional file 1: Appendix S2.

**Table 1:** Characteristics of included studies for incident heart failure outcomes in patients with a reported ejection fraction ≥ 50%

Study	Year	Country	Design	Population	Sample Size	Mean age (years)	Sex (% females)	% of patients with LVEF ≥ 50%	Follow-up (years)
Masoudi et al. [20]	2005	USA	Observational, retrospective cohort	T2DM, age ≥ 65 years, hospitalisation with HF	13,930	76	58	23	1.0
Romero et al. [21]	2011	Spain	Observational, prospective cohort	New-onset T2DM, HF	1519	72	54	51	4.7
Facila et al. [19]	2017	Spain	Observational, retrospective cohort	T2DM, discharged with diagnosis of acute decompensated HF	835	72	49	49	2.4
Aguilar et al. [18]	2011	USA	Observational, retrospective cohort	T2DM, prior diagnosis of HF	6185	68	7	45^a^	2

*T2DM* type 2 diabetes mellitus, *HF* heart failure, *USA* United States of America, *LVEF* left ventricular ejection fraction

^a^Study reported percentage of patients with LVEF≥ 40%

### Study characteristics

Study characteristics, baseline patient data and mortality outcomes are reported in Tables 1, 2 and 3. Populations studied were primarily in the United States of America and Spain. A total of 22, 469 individuals with HF were analysed with 7655 mortality events identified. Of the total number of individuals 10,168 (45%) had a preserved EF, however, one study reported the total proportion of participants with an EF ≥40% [18]. Follow-up ranged from 1 to 4.7 years. All studies used multiple covariates in adjustments of the effect size (Table 3) with two studies performing propensity matching in the analysis [18, 21].

**Table 2:** Participant characteristics of studies included in the meta-analysis

	Study	Mean age (years)	Female gender n (%)	EF ≥ 50% n (%)	CAD (%)	HTN (%)	PVD (%)	ACEi/ARB (%)	Beta-blocker (%)	Insulin (%)	Sulfonylurea (%)
Metformin treatment	Masoudi [20] (n = 1861)	75.8	1060 (57)	447 (24)	1135 (61)	1321 (71)	56 (3)	1191 (64)	707 (38)	354 (19)	1042 (56)
	Romero [21] (n = 926)	70.6	500 (54)	478 (52)	349 (38)	555 (60)	141 (15)	751 (81)	324 (35)	409 (44)	185 (20)
	Facila [19] (n = 275)	71.0	120 (44)	144 (52)	115 (42)	159 (58)	22 (8)	207 (75)	179 (65)	65 (24)	71 (26)
	Aguilar [18] (n = 1561)	67.3	122 (8)	727 (47)	514 (33)	1202 (77)	312 (20)	1350 (87)	929 (60)	523 (34)	923 (59)
	Pooled (n = 4623)	71.2±4	1802 (39)	1796 (39)	2113 (46)	3237 (70)	531 (11)	3499 (76)	2139 (46)	1351 (29)	2221 (48)
Non-Metformin treatment	Masoudi [20] (n = 12,069)	77.0	7000 (58)	2776 (23)	7966 (66)	8448 (70)	482 (4)	6879 (57)	3862 (32)	6638 (55)	6155 (51)
	Romero [21] (n = 593)	72.3	317 (54)	302 (51)	218 (37)	335 (57)	98 (17)	480 (81)	218 (37)	394 (66)	257 (43)
	Facila [19] (n = 560)	74.0	291 (52)	264 (47)	278 (50)	475 (85)	95 (17)	386 (69)	329 (59)	278 (49)	117 (21)
	Aguilar [18] (n = 4624)	70.0	282 (6)	2048 (44)	1665 (36)	3648 (75)	1147 (25)	3801 (82)	2839 (61)	2452 (53)	2719 (59)
	Pooled (n = 17,846)	73.3±3	7890 (44)	5390 (30)	10,127 (57)	12,906 (72)	1822 (10)	11,546 (65)	7248 (41)	9762 (55)	9248 (52)

*EF* ejection fraction, *CAD* coronary artery disease, *HTN* hypertension, *PVD* peripheral vascular disease, *ACEi* angiotensin converting enzyme inhibitor, *ARB* angiotensin receptor blocker

**Table 3:** Development of HF in patients with and without preserved EF

Study	Treatment	Total mortality events	Adjusted effect size [95% CI]	Covariates in effect size adjustment
Masoudi et al. [20]	Metformin vs. No Insulin sensitiser	4805	HR 0.86 [0.77, 0.97]	Patient factors (such as age and gender), treating physician and hospital characteristics
Romero et al. [21]	Metformin vs. No metformin	1045	HR 0.88 [0.83, 0.93]	Propensity matched: age, gender, educational level, social situation, occupation situation, type of HF, aetiology of HF, clinical signs, radiological data, ECG data, comorbidity, bloods (lipids, albumin, creatinine, eGFR, ACR, Hb, electrolyte levels), medication received, time to follow-up, place of diagnosis, income, early readmissions, time of hospitalisation, visits to medical professionals
Facila et al. [19]	Metformin vs. No metformin	382	HR 0.68 [0.53, 0.87]	Age, gender, prior admission for AHF, prior history of stroke, PVD, HTN, QRS > 120 ms, LVEF, eGFR, Hb, NT pro-BNP, treatment with beta-blockers
Aguilar et al. [18]	Metformin vs. No Metformin	1423	HR 0.76 [0.63, 0.92]	Propensity matched: Age, gender, race, BMI, SBP, DBP, LVEF, history of hypertension, PVD, CVD, AF, MI, cancer, COPD and diabetic complications, HF hospitalisation within the last 2 years, bloods (Hb, HbA1c, sodium, urea, eGFR, total cholesterol and triglycerides) and treatment with beta-blockers, sulfonylurea, thiazolidinediones, insulin, statins, ACEi/ARB

*HF* heart failure, *ECG* electrocardiogram, *eGFR* estimated glomerular filtration rate, *ACR* albumin-creatinine ration, *Hb* haemoglobin, *AHF* acute heart failure, *PVD* peripheral vascular disease, *HTN* hypertension, *LVEF* left ventricular ejection fraction, *NT pro-BNP* N-terminal pro b-type natriuretic peptide, *BMI* body mass index, *SBP* systolic blood pressure, *DBP* diastolic blood pressure, *CVD* cerebrovascular disease, *AF* atrial fibrillation, *MI* myocardial infarction, *COPD* chronic obstructive pulmonary disease, *ACEi* angiotensin converting enzyme, *ARB* angiotensin receptor blocker

### Risk of bias and quality assessments

All studies included were observational, retrospective cohort studies (Table 1). Based on Newcastle–Ottawa quality assessment, all studies were deemed high quality (Additional file 1: Appendix S3).

### Patient characteristics

Overall, patients in the metformin group tended to be younger than those in the non-metformin group (age 71.2 ± 4 years vs. 73.3 ± 3 years, respectively; Table 2). In the metformin group, 1796 (39%) of patients were female, 3499 (76%) were on an ACEi or ARB and 1351 (29%) were on insulin therapy. In contrast, in the non-metformin group 7890 (44%) were female, 11,546 (65%) were on an ACEi or ARB and 9762 (55%) were on insulin therapy.

### Mortality outcomes

**Fig. 2:**
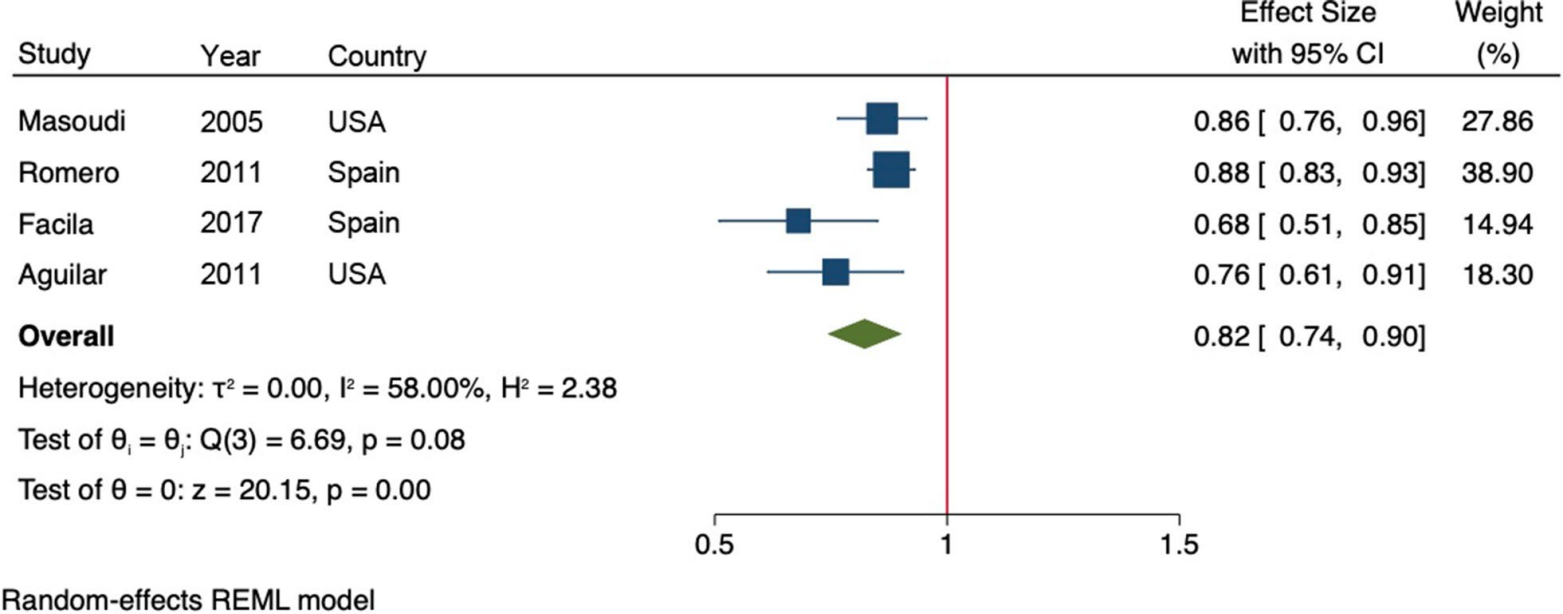
Meta-analysis of effect estimates for mortality in heart failure patients treated with and metformin therapy

The pooled HR for mortality in all HF individuals treated with metformin therapy was 0.82 (95% CI 0.74, 0.90, *p *< 0.001; Fig. 2). The summary estimate demonstrated a moderate degree of heterogeneity between studies (I^2^ = 58.0%, *p *< 0.001).

In the subgroup analysis (Table 4), metformin reduced mortality in both reduced and preserved EF after adjusting for concurrent treatment with cardio-protective medications, such as ACEi/ARB and beta-blocker therapy (β = − 0.2 [95% CI − 0.3, − 0.1], *p *= 0.02). After adjusting for the benefit of metformin in the EF ≥50% group irrespective of cardio-protective therapies, there was a significantly greater reduction in mortality (β = − 2.3 [95% CI − 3.3, − 1.3], *p *= 0.003). In the reduced EF sub-analysis, metformin was not associated with a mortality benefit (β = 0.2 [95% CI − 0.3, 0.6], p = 0.28). Furthermore, treatment with cardio-protective medications or insulin alone was associated with mortality reduction (ACEi/ARB *p *< 0.001; beta-blocker *p *= 0.017; insulin *p *= 0.002). Interestingly, female gender was associated with worse outcomes (*p *< 0.001).

**Table 4:** Interaction between metformin and subgroups on mortality outcomes

Subgroups	β-coefficient for the interaction with metformin and mortality [95% CI]	P-value	R-squared
Female Gender	4.9 [3.7–6.1]	< 0.001	0.993
LVEF≥50%	− 2.3 [− 3.3, − 1.3]	0.003	0.954
LVEF < 50%	0.2 [− 0.2, 0.6]	0.279	0.863
CAD	− 2.0 [− 10.3, 6.2]	0.530	0.801
PVD	− 3.4 [− 4.4, − 2.3]	0.001	0.969
Hypertension	− 1.4 [− 2.8, 0.1]	0.060	0.909
ACEi/ARB	− 1.1 [− 1.3, − 0.8]	< 0.001	0.974
Beta blocker	− 1.6 [− 2.7, − 0.5]	0.017	0.934
Insulin	− 1.8 [− 2.5, − 1.0]	0.002	0.958
Sulfonylurea	2.7 [− 0.5, 5.9]	0.077	0.915
Metformin treatment adjusted for LVEF, ACEi/ARB and beta-blocker therapy	− 0.2 [− 0.3, − 0.1]	0.020	0.996

*CI* confidence interval, *LVEF* left ventricular ejection fraction, *CAD* coronary artery disease, *PVD* peripheral vascular disease, *ACEi* angiotensin converting enzyme inhibitor, *ARB* angiotensin receptor blocker

## Discussion

This review demonstrates that metformin is associated with an 18% mortality reduction in all HF patients and that this benefit is observed in patients treated with concurrent cardio-protective medications, as seen in other clinical trials. However, this meta-analysis is the first to examine a mortality benefit of metformin therapy specifically in patients with a preserved EF.

### Diabetic cardiomyopathy

T2DM is a complex metabolic disorder, with the initial hallmarks of insulin resistance and progressive impairment in insulin secretion from the pancreas [28]. Over time a pro-inflammatory state develops, potentiated by alterations in gut microbiota and excess adiposity [29]. Ultimately, end-organ failure ensues.

Diabetic cardiomyopathy is a major adverse outcome of the disease. Where the pathophysiology of DM and atherosclerotic CAD is well understood [30], there is an emergence of data on the cellular and metabolic mechanisms of non-ischaemic driven diabetic cardiomyopathy. Alterations in the AMPK pathway and mitochondrial dysfunction are major components in the development of myocardial impairment [10].

### Metformin systemic and myocardial mechanism of action

Metformin is the most commonly prescribed anti-diabetic drug [31]. It has negligible hypoglycaemic risk, has beneficial effects on HbA1c and weight reduction and is relatively inexpensive [31]. In recent years its position in guideline-directed management of new-onset T2DM has somewhat changed. The American Diabetes Association still recommends metformin as first-line therapy in all newly diagnosed T2DM patients [31]; however, the European Society of Cardiology now recommends metformin as first-line therapy only in patients who are deemed not at high-risk or do not have established cardiovascular disease, instead recommending a sodium glucose cotransporter-2 inhibitor (SGLT-2i) or glucagon-like protein-1 receptor agonist (GLP-1 RA) for these patients [32].

In recent years our understanding of metformin’s mechanism of action has evolved. At the cellular level, metformin accumulates in the mitochondrial matrix ultimately causing a reduction in the synthesis of adenosine triphosphate (ATP) and an increase in the level of AMP resulting in the activation of the AMPK pathway [10]. In the liver, decreased ATP availability and inhibition of enzymes involved in lactate uptake results in inhibition of gluconeogenesis [9, 10, 33, 34]. Additionally, by activating AMPK metformin modifies lipid production and breakdown [10, 34]. In the intestinal tract, metformin inhibits glucose absorption and improves insulin production by the incretin affect [35]. In adipocyte tissue metformin reduces free-fatty acid release [36], further improving glucose uptake in other tissues such as skeletal muscle [37].

In the myocardium, metformin also activates AMPK resulting in increased glucose uptake, reduction in protein synthesis and improved mitochondrial function [10]. Furthermore, metformin decreases nitric oxide (NO) production via inhibition of inducible NO synthetase (iNOS) [12]. Finally, metformin has been shown to reduce collagen synthesis and fibrosis in myocardial tissue [14].

### Metformin in T2DM and cardiovascular disease

In the UK Prospective Diabetes Study, obese DM-individuals treated with metformin had a 42% (*p *= 0.017) risk reduction in diabetes-related deaths and a 36% (*p *= 0.011) reduction in all-cause mortality [38]. Furthermore, there was a 30% (*p *= 0.020) reduction of all macrovascular complications (MI, sudden death, angina, stroke and PVD) with metformin treatment [38].

The benefit of metformin therapy has been observed across multiple cardiovascular subgroups. In patients with established CAD, metformin was associated with a 29% reduction in cardiovascular mortality (HR 0.81 [95% CI 0.79, 0.84], *p *< 0.001) and a 33% reduction in all-cause mortality (HR 0.67 [95% CI 0.60, 0.75], *p *< 0.001) [39]. Furthermore, in a propensity-matched study of patients followed up after an acute coronary syndrome (ACS), metformin was associated with a 50% reduction (HR 0.50 [95% CI 0.26, 0.95], *p *= 0.035) in all-cause mortality [40]. However, in an analysis of patients treated with metformin presenting with their first ACS, metformin was associated with increased MACE (HR 1.13 [95% CI 1.03, 1.23], *p *= 0.006) but in those patients who survived beyond 30-days after the index event, metformin was not associated with MACE (HR 1.06 [95% CI 0.95, 1.17, *p *= 0.305) [41].

In heart transplant recipients, cardiac allograft vasculopathy (CAV) is a major cause of morbidity and mortality with limited treatment options [42]. However, in heart transplant patients treated with metformin prior to the development of CAV, metformin therapy was associated a 90% risk reduction in the development of CAV over a 20-year follow-up period (HR 0.1 [0.02, 0.46], p = 0.003) [42]. Furthermore, metformin was independently associated with a 91% reduction (p = 0.003) in the combined end-point of CAV and cardiovascular mortality in these patients [42].

### Metformin in HF

Historically, the use of metformin has been restricted in HF owing to concerns regarding the development of life-threatening lactic acidosis [43]. This adverse side-effect was largely extrapolated from data regarding phenformin, a biguanide that was ultimately withdrawn from the market [43]. However, the development of metformin-associated lactic acidosis in HF patients has since been refuted. In a large observational study spanning over 10 years, 27% of HF patients were on metformin therapy and there were no observed hospitalisations or deaths due to lactic acidosis [23].

Nonetheless, several observational studies have shown both morbidity and mortality benefits of HF patients treated with metformin. In a previous meta-analysis, metformin in HF patients was associated with a 7% reduction in HF hospitalisations (adjusted RR 0.93 [95% CI 0.86, 0.98], *p *= 0.01) and a 20% reduction in all-cause mortality (RR 0.80 [95% CI 0.74, 0.87], *p *< 0.001) [16]. Furthermore, this benefit extends to subgroups of HF such that in patients with CAD, all-cause mortality was reduced by 16% (HR 0.84 [0.81, 0.87], *p *= 0.03) [39]. In patients with hypertension, long-term metformin treatment was associated with a reduction in LV filling pressures and LV mass over-time [44]. In these patients the incidence of symptomatic HFpEF was also reduced with metformin therapy compared to non-metformin therapy (4.6% vs. 11.9% respectively, *p *= 0.020) [44]. The results of the current meta-analysis supporting these findings in the HF population with an 18% reduction in mortality associated with metformin therapy. Furthermore, this meta-analysis has shown a mortality benefit associated with metformin in patients with HFpEF. This is of clinical relevance as there are limited therapeutic options with mortality benefits in this population of HF patients.

### Limitations

Despite a thorough literature search, there is a potential risk of not identifying all studies that have evaluated metformin use in HF patients, particularly those that were performed as a sub-group analysis. However, our use of reference mining and grey-literature searches are likely to have minimised this. Furthermore, due to the nature of observational studies, unaccounted confounding variables may have influenced individual study results. No randomised control trial of the use of metformin in HF patients exists and so this issue cannot be mitigated. The use of aggregate data rather than individual patient data may have limited the analysis.

Finally, our analysis was limited to observational studies of metformin in all HF patients, rather than HFpEF as a single entity. Due to the lack of reporting of outcomes in HFpEF patients our analysis was limited to a meta-regression in this sub-group. Furthermore, due to the low number of studies included we encountered a significant amount of heterogeneity, ultimately reflecting the paucity of evidence in this area and the requirement for further research.

## Conclusions

This meta-analysis is the first to highlight a mortality benefit for metformin therapy in HF patients with a preserved EF.

## Supplementary information

**Additional file 1: Appendix S1.** Search terms. **Appendix S2.** Studies that underwent full-text review and were excluded from the final analysis. **Appendix S3.** Quality assessment using the Newcastle-Ottawa quality assessment scale. * indicates the study has met the criteria.

## Data Availability

Made on request to the authors.

## Abbreviations

ACEi: Angiotensin converting enzyme inhibitors
AMP: Adenosine monophosphate
AMPK: Adenosine monophosphate-activated protein kinase
ARB: Angiotensin receptor blocker
ATP: Adenosine triphosphate
CAD: Coronary artery disease
CAV: Cardiac allograph vasculopathy
EF: Ejection fraction
GLP-1 RA: Glucagon like protein-1 receptor agonist
HR: Hazard ratios
HF: Heart failure
HFpEF: HF with preserved ejection fraction
HFrEF: HF with reduced ejection fraction
ICD: International Classification of Diseases
iNOS: Inducible nitric oxide synthetase
LV: left ventricular
MACE: Major adverse cardiovascular events
NO: Nitric oxide
PRISMA: Preferred Reporting Items for Systematic Reviews and Meta-Analysis
PVD: Peripheral vascular disease
SGLT-2i: Sodium glucose cotransporter-2 inhibitor
T2DM: Type 2 diabetes mellitus
