# Supplementary material for: Metformin treatment in heart failure with preserved ejection fraction: a systematic review and meta-regression analysis
Source: Cardiovasc Diabetol. 2020 Aug 5;19:124. doi: 10.1186/s12933-020-01100-w (PMC7409497; doi:10.1186/s12933-020-01100-w)
Supplement: Supplementary file 1 — Additional file 1: Appendix S1. Search terms. Appendix S2. Studies that underwent full-text review and were excluded from the final analysis. Appendix S3. Quality assessment using the Newcastle-Ottawa quality assessment scale. * indicates the study has met the criteria. [file 12933_2020_1100_MOESM1_ESM.docx]

**Additional file**

**Appendix S1.** Search terms

MEDLINE: ‘heart failure/’, ‘cardiomyopathies’, ‘heart failure, diastolic’, ‘heart failure’ adj ‘preserved ejection fraction’, ‘left ventricular hypertrophy’, ‘metformin/’, ‘biguanide/’, ‘mortality/’, ‘exercise capacity’

EMTREE: ‘heart failure/’, ‘heart or cardiac or myocardial’ adj ‘failure or decompensation or insufficient or incompetence’, diastolic heart failure/, ‘heart failure’ adj ‘preserved ejection fraction’, ‘left ventricular hypertrophy’, ‘metformin/’, ‘biguanide/’, ‘mortality’, ‘exercise capacity’

**Appendix S2.** Studies that underwent full-text review and were excluded from the final analysis.

| **Study** | **Year** | **Country** | **Design** | **Reason for exclusion** |
| --- | --- | --- | --- | --- |
| Shah et al.  (22) | 2010 | USA | Observational, retrospective cohort | Excluded HF patients with LVEF≥40%. |
| Andersson et al. (23) | 2010 | Denmark | Observational, retrospective cohort | HF diagnosis was based on ICD codes. Proportion of patients with LVEF≥50% was not reported. |
| Eurich et al.  (24) | 2005 | Canada | Observational, retrospective cohort | HF diagnosis was based on ICD codes. Proportion of patients with LVEF≥50% was not reported. |
| Evans et al.  (25) | 2010 | Scotland | Observational, retrospective cohort | HF diagnosis was based on ICD codes. Proportion of patients with LVEF≥50% was not reported. |
| MacDonald et al. (26) | 2010 | UK | Observational, nested case-control | HF diagnosis was based on ICD codes. Proportion of patients with LVEF≥50% was not reported. |
| Retwinski et al. (27) | 2018 | Poland | Observational, retrospective cohort | HF diagnosis was based on clinical judgement. Proportion of patients with LVEF≥50% was not reported. |

HF = heart failure; LVEF = left ventricular ejection fraction; ICD = International Classification of Diseases; USA = United States of America; UK = United Kingdom.

**Appendix S3**. Quality assessment using the Newcastle-Ottawa quality assessment scale. * indicates the study has met the criteria.

| **Study** | **Overall rating**  **(good/fair/low)** | **Selection** | | | | **Comparability** | **Outcome** | | |
| --- | --- | --- | --- | --- | --- | --- | --- | --- | --- |
|  |  | Representative-ness of the exposed cohort | Selection of the non-exposed group | Ascertainment of exposure | Demonstration that outcome of interest was not present at the start of the study | Comparability of cohorts on the basis of the design or analysis controlled for confounders | Assessment of outcome | Was follow-up long enough for outcomes to occur | Adequacy of follow-up of cohorts |
| Masoudi | Good | * | * | * | * | ** | * | * | * |
| Romero | Good | * | * | * | * | ** | * | * | * |
| Facila | Good | * | * | * | * | ** | * | * | * |
| Aguilar | Good | * | * | * | * | ** | * | * | * |
